# Occupational Exposure to Metal Engineered Nanoparticles: A Human Biomonitoring Pilot Study Involving Italian Nanomaterial Workers

**DOI:** 10.3390/toxics11020120

**Published:** 2023-01-26

**Authors:** Beatrice Bocca, Beatrice Battistini, Veruscka Leso, Luca Fontana, Stefano Caimi, Mauro Fedele, Ivo Iavicoli

**Affiliations:** 1 Department of Environment and Health, Istituto Superiore di Sanità, 00161 Rome, Italy; 2 Department of Public Health, University of Naples Federico II, 80138 Naples, Italy

**Keywords:** nanomaterials, exposure assessment, biological monitoring, biomarkers, risk assessment, risk management

## Abstract

Advances in nanotechnology have led to an increased use of engineered nanoparticles (ENPs) and the likelihood for occupational exposures. However, how to assess such exposure remains a challenge. In this study, a methodology for human biomonitoring, based on Single Particle Inductively Coupled Plasma Mass Spectrometry (SP-ICP-MS), was developed as a tool to assess the ENPs exposure of workers involved in nanomaterial activities in two Italian Companies. The method was validated for size and number concentration determination of Ag, Au, In_2_O_3_, Ir, Pd, Pt, and TiO_2_ NPs in urine and blood samples. The results showed the presence of In_2_O_3_ NPs in blood of exposed workers (mean, 38 nm and 10,371 particles/mL), but not in blood of controls. Silver, Au, and TiO_2_ NPs were found in urine (mean, Ag 29 nm and 16,568 particles/mL) or blood (mean, Au 15 nm and 126,635 particles/mL; TiO_2_ 84 nm and 27,705 particles/mL) of workers, though these NPs were found also in controls. The presence of ENPs in both workers and controls suggested that the extra-professional exposure is a source of ENPs that cannot be disregarded. Iridium, Pd, and Pt NPs were not detected neither in blood nor in urine. Overall, the findings provided a rational basis to evaluate the exposure assessment to ENPs in cohorts of workers as part of risk assessment and risk management processes in workplaces.

## 1. Introduction

The nanotechnology, intended as a system of innovative methods to control and manipulate matter at near-atomic scale to produce new materials, structures, and devices [1], is driving promising advances in science, industry and commerce [2]. The increasing worldwide use of engineered nanoparticles (ENPs) in products is inevitably resulting in enhanced exposure of both consumers and workers [3]. The latter are being potentially exposed in research laboratories, start-up companies, production facilities, and operations where NPs are processed, used, disposed or recycled [1].

However, despite the extensive production and use of nano-products, our knowledge concerning NPs health and safety issues is still in a developing phase, and also the assessment of risks derived from NPs exposure remains a challenging task [4]. This is related to the difficulties in characterizing NPs potential hazards due to their various physico-chemical properties, as well as in assessing exposure levels through both environmental and human biological monitoring (HBM) measures. Concerning this latter aspect, although HBM is a suitable means to achieve an appropriate evaluation of the “personal” exposure experienced by workers, no instances or regulatory requirements for its routinely application in occupational settings have been developed.

Concerning the internal doses addressed in NPs occupationally exposed subjects through HBM, preliminary and fragmented data are available. Previous studies were based on the determination of total metal content (dissolved plus nanoforms) in blood and urine of workers who manufactured Ag, In, and Mo nanomaterials [5,6]. Only one study demonstrated an increased Ti concentration and the presence of ≥100 nm sized TiO_2_-crystals in the exhaled breath condensate (EBC) samples collected from TiO_2_ NPs production workers [7]. In the same population, urinary Ti concentrations were under the limit of quantification and no crystals could be detected. Additionally, in airport employees, operating on the apron, nearby airplane parking positions, and exposed via inhalation to incidental ultrafine particles generated by jet engines a sparse population of ca. 500 nm sized particles in the EBC samples were determined and concentrations of metals as Al, Cd, and Cr were detected in 19%, 22%, and 79% of all subjects, respectively [8]. More recently, our group developed an analytical protocol using the Single Particle-Inductively Coupled Plasma Mass Spectrometry (SP-ICP-MS) to determine the metal-oxide NPs in various HBM matrices—as EBC, plasma, and urine—of welders exposed to incidental ultrafine particles emitted during welding operations [9]. We could demonstrate significantly higher median Cr_2_O_3_ NPs concentration in the EBC compared to the plasma samples of welders, with a significant difference between pre- and post-shift samples, these latter showing significantly higher levels.

Based on the experience and knowledge gained during our first study, the present investigation explores the potential applicability of the SP-ICP-MS technique for the HBM of workers exposed to ENPs in different exposure scenarios. In particular, we assessed, through biological monitoring analyses, the exposure to ENPs in a selected sample of workers engaged in a nanotechnology research laboratory (Company A) and in workers of a chemical facility where colloidal NPs of polyvinylpyrrolidone (PVP)-Pt are synthesized and precious metals at the nanoscale are used for the production of catalytic converters (Company B). Overall, this may provide interesting guidance to future investigations aimed to achieve knowledge advances able to inform suitable risk assessment and management procedures in ENPs-related occupations.

## 2. Materials and Methods

### 2.1. Population Recruitment, Biological Samples Collection and Preparation

Workers employed in Company A and B for at least 6 months and involved in nanomaterial related activities were considered eligible to be enrolled in the study. Employees from the same companies, but not involved in nanomaterial related tasks, were considered suitable to be included as unexposed controls. The sample population recruitment was performed on a voluntary basis from February to March 2019 and all the employees were required to provide written informed consent to participate in the study. Only workers affected by acute or chronic diseases potentially interfering with HBM results or unwilling to provide consent were excluded from the research. A screening form was employed to obtain information on the length of employment, administrative work organization, characteristics of the performed tasks, as well as collective and individual preventive measures adopted. The study protocol was reviewed and approved by the Ethics Committee of the Istituto Superiore di Sanità, Rome, Italy (Prot. PRE 99/18).

Urine and blood samples from workers of the two different companies were collected during one working week. Urine samples from exposed employees were sampled at the beginning and at the end of the shift of 4 workweek days (1st–4th day) and collected in high density 100 mL polyethylene bottles (Kartell, Milan, Italy) previously decontaminated with 10% ultrapure HNO_3_ (Normatom, Leuven, Belgium). Approximately 6 mL of blood were collected from each worker at the end of the working week (4th day). The blood withdrawal was carried out using metal free containers (K-EDTA vacutainer BD tubes; Becton Dickinson Labware, Franklin Lakes, NJ, USA) and metal free needles (Becton Dickinson Labware) to reduce the risk of metal contamination. Biological samples were immediately stored at −20 °C until analysis. Controls provided samples at the same time points of exposed workers.

Urine and blood samples were thawed at room temperature and shaken before use. Then, one mL of urine was diluted 1:10 with ultrapure deionized water (Milli-Q Element, Bedford, MA, USA). One mL of blood was subjected to alkaline extraction with the addition of 3.5 mL of 25% *v*/*v* tetramethylammonium hydroxide (TMAH) (Sigma-Aldrich, St. Louis, MO, USA); the sample was then sonicated for 1 h in an ice-cooled water bath and left for 24 h at room temperature. At full extraction, 0.5 mL of a 0.1% *v*/*v* Triton-X solution (Alfa Aesar, Ward Hill, MA, USA) was added [10]. Blank samples (ultrapure deionized water or TMAH water solution) were prepared following the same procedure as samples.

### 2.2. Certified NPs Reference Standards

The following metal-based NPs certified references standards (nominal size; concentration) were used for this study: Ag (40 nm; 0.02 mg/mL), Au (60 nm; 1.9 × 10^10^ particles/mL) and TiO_2_ (<100 nm; 480–520 mg/mL) were purchased from Sigma-Aldrich; while In_2_O_3_ (20–70 nm; 200 mg/mL), Ir (15 nm; 1 mg/mL), Pd (15 nm; 1 mg/mL) and Pt (15 nm; 1 mg/mL) from US Research Nano-materials Inc. (Houston, TX, USA). Each standard was progressively diluted with ultrapure deionized water. After each dilution step, the suspensions of NPs standards were stirred on vortex for 1 min and sonicated for 10 min using an ultrasonic ice-cooled water bath to ensure the homogeneous dispersion of NPs and prevent any agglomerated particle clusters. These standard suspensions were spiked to pooled urine and blood samples to build the calibration curves and validate the whole analytical procedure.

### 2.3. SP-ICP-MS Analysis of NPs

Biological samples were analyzed by the iCAP Q inductively Coupled Plasma Mass Spectrometry (ICP-MS) in Single Particle (SP) mode (Thermo Fisher, Bremen, Germany) to detect the exposure of workers and controls to NPs of metals. The instrumental and method parameters used are reported in Table 1. To calculate the sensitivity of the ICP-MS system, single-element stock solutions were used (CPAChem, C.P.A. Ltd., Stara Zagora, Bulgaria) at the analytical concentration of 1 µg/L. To evaluate the transport efficiency of the ICP-MS sample introduction system replicated measurements were performed on Au NP reference standard at nominal size of 60 nm and concentration of 19,000 particles/mL [10,11,12,13]. The isotopes ^107^Ag, ^197^Au, ^115^In, ^193^Ir, ^106^Pd, ^195^Pt, and ^47^Ti were used for quantification; a dwell time of 5 msec and an analysis time of 60 s per sample were applied. The SP-ICP-MS allows to distinguish between the dissolved metals (background) and the metals in nanoform that appeared as signals (spikes) in the raw data. Each spike represents the single NP and the number of spikes per minute is proportional to the number of particles/mL, whilst the intensity of spikes in counts per second (cps) informs on the size of particles (in nm). The raw data were then converted in the particle size distribution (PSD) with the knowledge of the metal or metal-oxide density and by assuming the spherical form of particles. Mean particle diameter (in nm), particle size distribution (PSD in nm), and particle number concentration (as particles/mL) were generated by the Thermo Scientific™ Qtegra software.

The results of ENPs in urine are reported as mean values ± standard deviation (SD) of the diameter in nm, particle number in particles/mL and concentration in ng/mL detected in pre-shift and post-shift samples of 4 workweek days (1st–4th day). The results in blood are reported as mean values ± standard deviation (SD) of the diameter in nm, particle number in particles/mL and concentration in ng/mL detected at the end of working week (4th day).

### 2.4. Method Validation

The SP-ICP-MS method was validated according to the ISO/IEC 17025:2017 guideline [14]. Based on this standard, linearity, limit of detection (LoD), repeatability, and recovery must be determined. In particular, the standard addition procedure was used for calibration by adding known amounts of certified NPs in pooled urine and blood samples and the linearity range was expressed as the R^2^ of the calibration curves. The calibration curves allowed also to extrapolate the concentration of NPs (ng/mL or particles/mL for Au) in the samples of exposed workers and controls. To this end, pooled urine and blood samples were spiked with the following increasing concentrations of certified NPs reference standards: Ag 40 nm: 0.01, 0.05 and 0.1 ng/mL; Pt 15 nm: 0.1, 0.5 and 1.0 ng/mL; Pd 15 nm, In_2_O_3_ 20–70 nm, Ir 15 nm, and TiO_2_ < 100 nm: 1, 5 and 10 ng/mL; and Au 60 nm: 9500, 19,000 and 38,000 particles/mL.

The LoD in diameter (nm) was automatically generated by the software, but the minimum detectable size experimentally observed in the obtained PSDs was also evaluated for comparison. The LoD in concentration was calculated with the 3σ threshold criteria on 5 different measurements of matrices.

To calculate recovery and repeatability on both particle concentration and diameter the following spikes in each matrix were used: Ag 40 nm: 0.05 ng/mL; Pt 15 nm: 0.5 ng/mL; In_2_O_3_ 20–70 nm, Ir 15 nm, Pd 15 nm, and TiO_2_ < 100 nm: 5 ng/mL; and Au 60 nm: 19,000 particles/mL. Recovery on concentration was assessed by comparing the particles/mL observed in matrix analysis and the particles/mL observed in blank analysis. Recovery on size was assessed by the ratio between the size certified by the supplier and that found by the SP-ICP-MS analysis. Repeatability was expressed as the relative standard deviation (RSD%) of five repeated measurements of matrices over the SP-ICP-MS sequence.

## 3. Results

### 3.1. Investigated Population

The study population comprised three workers from the Company A (two males and one female) and three workers from Company B (2 males and 1 female). Workers from the Company A were researchers involved in the growth of InAs semiconductor nanostructures by chemical beam epitaxy (CBE) technique, using organometallic pre-cursors. In some cases, the growth was catalyzed by Au NPs. The mean (±SD) length of employment in workers of Company A was 4.3 ± 1.5 years and shift-works lasted from a minimum of 6 h to a maximum of 8, with work 5 days a week. In Company B, workers synthesized colloidal NPs of polyvinylpyrrolidone (PVP)-Pt in the presence of ascorbic acid (reducing agent). In the same Company precious metals as Ir, Pd, and Pt were used for the production of catalytic converters. Length of employment resulted in a mean ± SD of 8 ± 3.6 years, with shift-works lasting from 4 to 8 h, for 5 days a week. In both companies, the collective and personal protective equipment (PPE) routinely used included the following: chemical fume hood, chemical protective clothing and goggles; disposable gloves; facial mask class FFP3. Two male administrative workers from the Company A were enrolled as unexposed controls. They had been working in such Company for a mean ± SD of 14 ± 8.4 years, for 8 h a day, 5 days a week.

### 3.2. SP-ICP-MS Method Validation Performances in Urine and Blood

**Figure 1:**
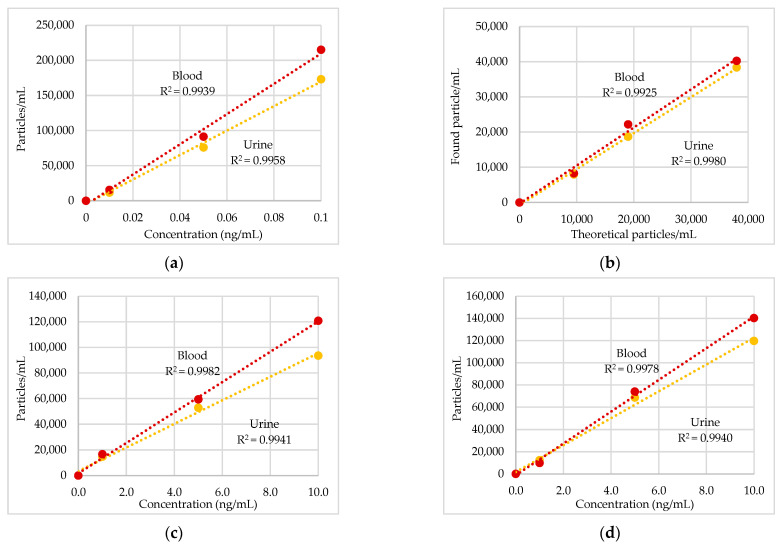

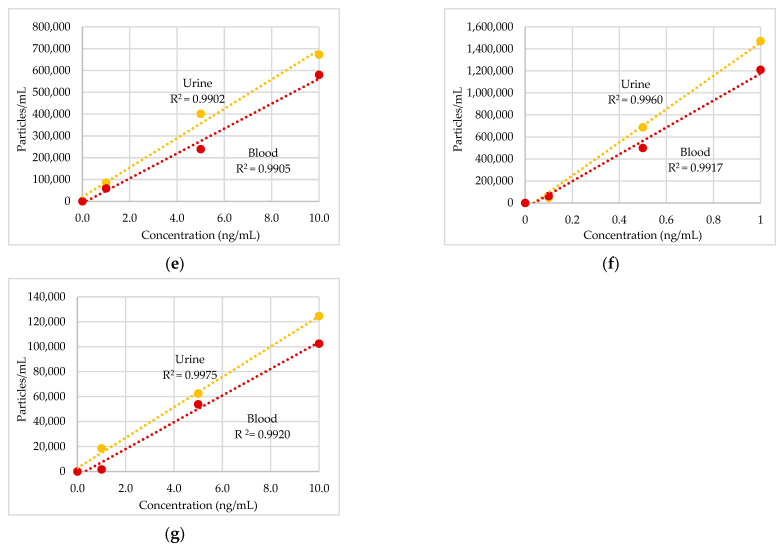
Calibration curves and linear dependence between particles/mL and theoretical concentrations of metal nanoparticles (NPs) reference standard: (**a**) Ag, 40 nm; (**b**) Au, 60 nm; (**c**) In_2_O_3_, 20–70 nm; (**d**) Ir, 15 nm; (**e**) Pd, 15 nm; (**f**) Pt, 15 nm; (**g**) TiO_2_ < 100 nm.

Figure 1 shows the linearity ranges expressed as the R^2^ values (0.990–0.998) between the theoretical particle concentrations and the number of particles measured by SP-ICP-MS. The SP-ICP-MS validation parameters in urine and blood are reported in Table 2. The LoD in concentration (ng/mL) in urine and blood, respectively, were as follows: Ag, 0.001 and 0.001; In_2_O_3_, 0.36 and 0.40; Ir, 0.76 and 1.02; Pd, 0.33 and 0.68; Pt, 0.07 and 0.13; TiO_2_, 0.28 and 0.81. The LoD for Au was 1063 particles/mL in urine and 1332 particles/mL in blood. The LoD in size (nm) in urine and blood, respectively, were as follows: Ag, 12 and 15; Au, 8 and 11; In_2_O_3_, 12 and 17; Ir, 7 and 8; Pd, 24 and 26; Pt, 16 and 17; TiO_2_, 45 and 50. Number particles recoveries were in the range 80–108% in urine and 87–106% in blood. The repeatability on particle concentration and diameter was lower than 8.7% in urine and 8.4% in blood.

### 3.3. Size and Concentration of Metal NPs in Urine and Blood

Urine and blood of three workers from the Company A, three workers from the Company B and two administrative workers as controls from the Company A were analysed by the validated SP-ICP-MS method.

Table 3 reports the results of the analysis of ENPs in urine of workers and controls at pre-shift (1st–4th day, mean ± SD) and post-shift (1st–4th day, mean ± SD). One worker from Company A (no. 3) showed Ag NPs in urine both at pre- and post-shift with mean diameter of ca. 29 nm and mean number of particles of 19,180 particles/mL (0.012 ng/mL) at pre-shift and 13,955 particles/mL (0.009 ng/mL) at post-shift. No NPs of Au, In_2_O_3_, Ir, Pd, Pt, and TiO_2_ were found in any exposed worker of either Company A or Company B. In controls, NPs of Ag were found with diameter of ca. 29 nm and mean number of particles of 7680 particles/mL 0.006 ng/mL) at pre-shift and 6630 particles/mL (0.006 ng/mL) at post-shift for one subject (no. 7) and 12,215 particles/mL (0.008 ng/mL) at pre-shift and 10,470 particles/mL (0.007 ng/mL) at post-shift for another subject (no. 8).

**Figure 2:**
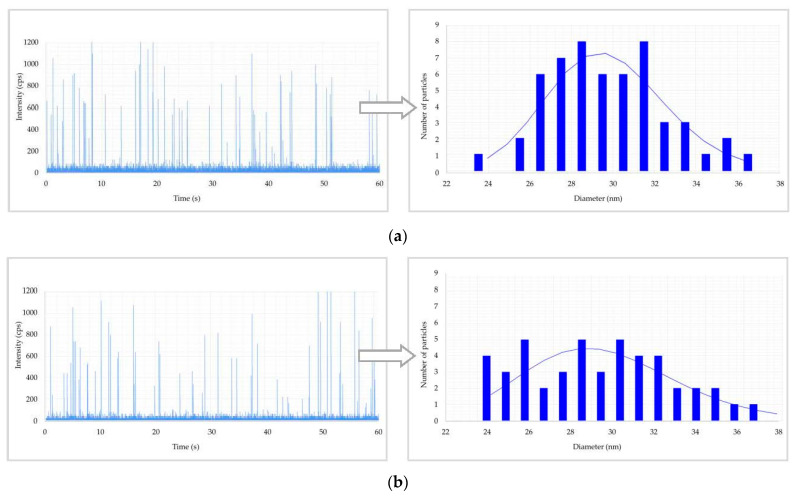

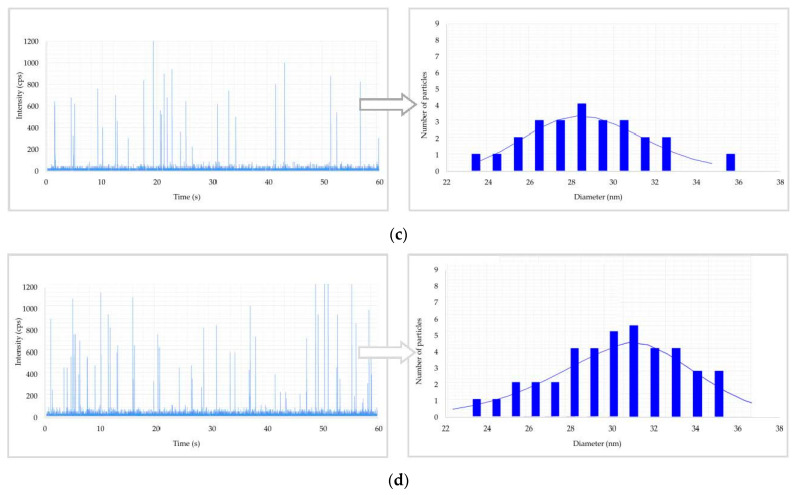
Results of Ag nanoparticles (NPs) found in urine of a worker: (**a**) raw data and particle size distribution (PSD) at pre-shift start of week; (**b**) raw data and PSD at post-shift start of week; (**c**) raw data and PSD at post shift end of week; (**d**) raw data and PSD in a control worker.

**Figure 3:**
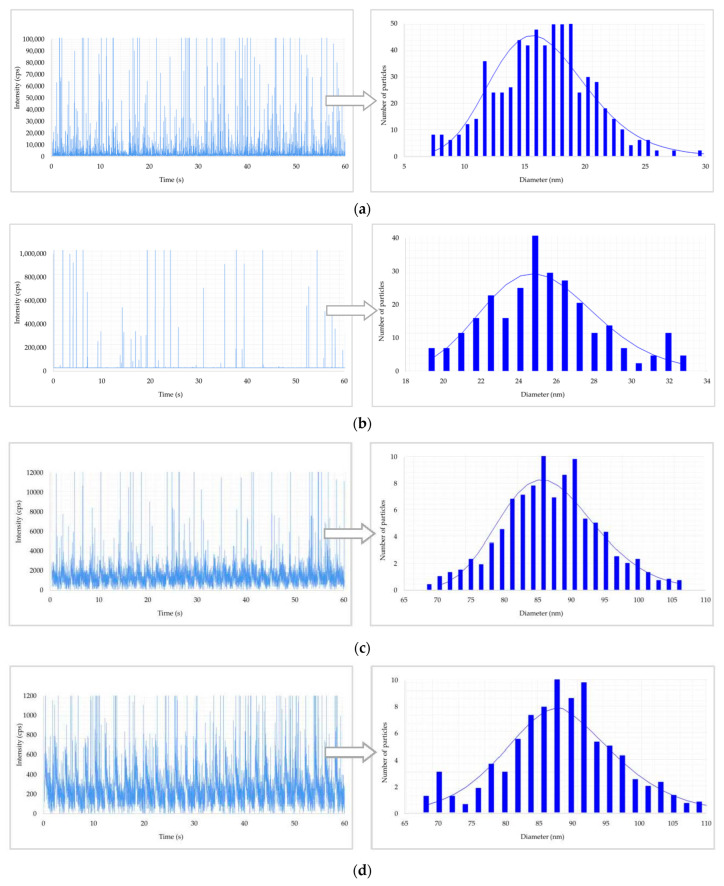
Results of metal nanoparticles (NPs) found in blood of a worker: (**a**) raw data and particle size distribution (PSD) of Au NPs at the post shift end of week; (**b**) raw data and PSD of In NPs at the post shift end of week; (**c**) raw data and PSD of Ti NPs at the post shift end of week; (**d**) raw data and PSD of Ti NPs in a control worker.

The raw data and PSD are reported in Figure 2 and Figure 3 for urine and blood, respectively. In these figures, when ENPs are present in samples, discontinuous signals in the form of spikes appeared in the raw data. Each spike represents the signal from a single NP. The number of spikes per min is proportional to the number of particles per mL, whilst the intensity (i.e., the height) of spikes (in cps) is proportional to the size of particles (in nm). So, the spikes with higher height in the raw data originated from bigger particles. Then, the particle signals are plotted in a PSD that represents the percentage of particles of a certain size present in samples. The maximum of the PSD indicates the most frequently detected size.

**Figure 4:**
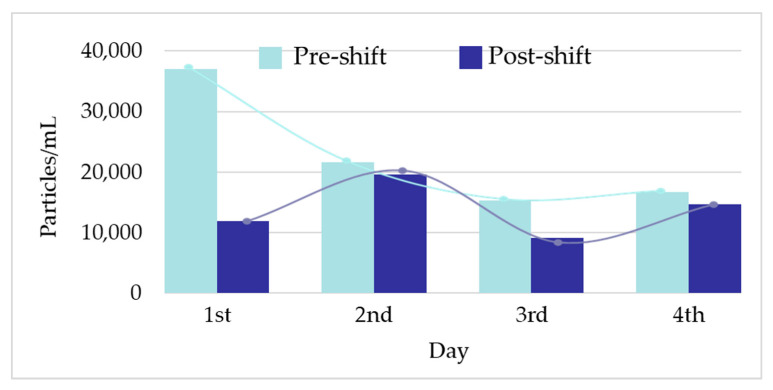
Workers urinary excretion profile of Ag nanoparticles (NPs) during one working week (1st–4th day).

**Figure 5:**
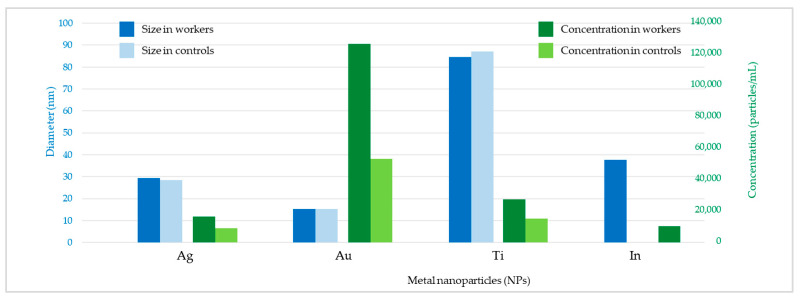
Mean diameter (nm) and number concentration (particles/mL) of metal nanoparticles (NPs) for workers and controls of the two companies.

Figure 2 shows the raw data and PSD of Ag NPs in urine of the worker (no. 3) from Company A during the working week analysed. In particular, the particle raw data and PSD at pre- (Figure 2a) and at post-shift (Figure 2b) of the first day of working week, at post-shift of the last day of working week (Figure 2c) and an example of raw data and PSD in a control subject (Figure 2d) are depicted. A time-dependent urine excretion of Ag NPs during the work week was reported for the worker (no. 3) from Company A as reported in Figure 4. The results of the analysis of metal NPs in blood of workers from Company A and B and controls, at the end of working week (4th day), are reported in Table 4. The Au NPs were observed in all the workers of Company A and in all controls with a comparable diameter of 15 nm and number of particles ranging from 62,794 to 251,177 particles/mL for workers and from 95,838 to 10,989 particles/mL for controls. In addition, TiO_2_ NPs were detected both in workers and controls, with a size between 82 nm and 90 nm and number of particles between 14,334 particles/mL and 49,092 particles/mL (1.65–4.90 ng/mL). The NPs of In_2_O_3_ were detected only in two workers from Company A (no. 2 and 3) and not in the control group. The size measured was ca. 38 nm and number of particles was 14,020 particles/mL (1.00 ng/mL) and 6722 particles/mL (0.41 ng/mL). No NPs were detected in blood of workers from Company B. Figure 3 reports the raw data and PSD for Au NPs (Figure 3a), In_2_O_3_ NPs (Figure 3b) and TiO_2_ NPs (Figure 3c,d) detected in blood of workers and controls at the end of week. Figure 5 summarizes the particles size and concentrations found in blood and urine of workers and controls at the two Companies.

## 4. Discussion

This HBM pilot study aimed to evaluate the occupational exposure to metal ENPs in workers involved in nanomaterials production in two Italian Companies (A and B). Although the limited number of workers enrolled in the study, to our knowledge, this is the first attempt to assess, in field, the exposure to metal ENPs using the SP-ICP-MS in the analysis of HBM samples. This protocol was firstly employed by our group of research to assess the exposure to incidental metal-oxide NPs emitted during welding operations. It offered the opportunity to determine both the size and number concentration of Cr_2_O_3_, Mn_3_O_4_ and NiO NPs in different biological matrices (EBC, plasma and urine) of stainless-steel welders workers [9]. The same technique was used here for the HBM of workers exposed to ENPs such as Ag, Au, In_2_O_3_, Ir, Pd, Pt, and TiO_2_ in two different occupational exposure scenarios. The SP-ICP-MS technique in urine and blood was validated according to ISO/IEC 17025:2017 [14]. The linearly concentration range (Figure 1) was between 0.01 and 0.1 ng/mL (Ag NPs), 0.1 and 1.0 ng/mL (Pt NPs), 1 and 10 ng/mL (Pd, In_2_O_3_, Ir and TiO_2_ NPs) and 9500 and 38,000 particles/mL (Au NPs) in both matrices and the R^2^ values observed were >0.99 for all the metal NPs. Table 2 shows the LoDs in concentration ranging from 0.001 ng/mL (Ag NPs) to 0.40 ng/mL (In_2_O_3_ NPs) in urine and 0.001 ng/mL (Ag NPs) and 1.02 ng/mL (Ir NPs) in blood; whilst, the LoDs in size ranged from ca. 7 nm (Ir NPs) to ca. 50 nm (TiO_2_ NPs) in urine and blood respectively. Accurate recoveries on concentration (80–108%) were obtained, and repeatability on particle counting was better than 6% both in urine and blood (Table 2). Good agreement was found between the mean size of NPs standards observed in both matrices and the certified size, except for Ir and Pd NPs that measured ca. 30 nm instead of 15 nm certified. The method resulted fast (60 s of analysis for sample), required minimum quantity of specimen and limited sample preparation (without preconcentration step) and was able to detect very low ENP concentrations. Thus, the method can be recommended for the reliable and routine measurements of ENPs in field surveys.

Regarding the ENPs analysis in samples, in Company A, Ag was detected in urine in only one out of three exposed workers with a mean size of 29 nm at pre- and post-shift and mean concentration of 19,180 particles/mL (0.012 ng/mL) at pre-shift and 13,955 particles/mL (0.009 ng/mL) at post-shift (Table 3). Interestingly, all the two subjects enrolled as controls had comparable size of Ag (mean, 29 nm pre- and post-shift) but a lower number concentration of particles (mean, 9948 particles/mL at pre-shift and 8550 particles/mL at post-shift) compared to those determined in the exposed worker (Table 3). This finding, on the one side, indicated the general low levels of occupational exposure to Ag in Company A (Figure 2a–c) because the exposed employees had concentrations comparable to those found in unexposed controls (Figure 2d). On the other side, it may suggest a possible extra-professional source of exposure to Ag NPs. To this regard, Ag NPs are widely used in consumer products such as cosmetics, textiles, health-care products [15], and in the food industry [16]. Additionally, the lack of any increase in the Ag NPs concentration and particle counting in urine samples collected at pre- and post-shift might further confirm the role of extra-professional sources of exposure in affecting the HBM results (Figure 4). The origin of Ag NPs in controls is questionable considering that this kind of NPs are also used in dental resin composites, cleaning and disinfectant products and water filters, with their possible release and absorption by human organism [17]. Lee et al. [5] showed a level of Ag of 0.043 μg/dL and not detectable concentrations in two workers involved in the manufacturing of Ag nanomaterials.

Studies on Ag NPs orally administered to rats, showed that these NPs were distributed in all tissues and the concentration was dependent on the dimensions and the exposure time. In fact, short treatment duration led to higher concentrations in duodenum, the first contact site [18]; while for a longer exposure time, the NPs were absorbed and transported through the blood up to different organs, such as liver and spleen and, finally, to the kidneys for excretion [19]. Additionally, the particle size has been also demonstrated as an influencing factor for the glomerular filtration of the particles themselves. In fact, <6 nm sized particles are typically filtered and excreted via the renal system, while those >8 nm are primarily eliminated via the hepatobiliary system as they are not normally filtered by the renal glomerulus [20]. No other metal ENPs analysed were found in urine samples collected from both exposed employees and unexposed subjects (Table 3). Similarly, no NPs of Cr_2_O_3_, Mn_3_O_4_ and NiO [9] and TiO_2_ [7] were observed in urine of stainless-steel welders exposed to metal-oxide NPs.

Comparable counts and diameters of Au NPs (mean, 15 nm, 97,346 particles/mL) and TiO_2_ (mean, 85 nm, 22,739 particles/mL, 2.4 ng/mL) were observed in blood of workers and controls of Company A at the end of the working week (Table 4). As for Ag, these data may be explained by a possible general exposure to Au and TiO_2_ NPs by daily-life sources. In this view, different previous papers observed human exposure after the skin contact with Au and TiO_2_ NPs present in consumer products [12,21] or through the ingestion of food products containing TiO_2_ NP-additives [22]. In addition, Au NPs could originate from the cigarette smoke or from dental alloy [17]. Pelclova et al. [23] found TiO_2_ NPs after sunscreen application in blood and urine of volunteers up to one week. The human exposure of the general population and the systemic uptake of TiO_2_ NPs was also observed by Peters et al. [24] and Heringa et al., [25] in post-mortem tissues (liver, spleen, kidney, jejunum, and ileum). In other occupational settings the exposure to ENPs as TiO_2_ were effectively reduced by proper local exhaust ventilation (LEV), filtration, containment, and good work practices [26].

To the opposite of blood, Au NPs and TiO_2_ NPs were not detected in urine neither in exposed nor in unexposed subjects. Other investigations reported that Au NPs were mainly excreted in the feces rather than in urine, due to the mucociliary escalator clearing mechanism and the involvement of the gastrointestinal tract [27]. The same authors observed that Au NPs translocated into the systemic circulation and could be detected in the blood and heart. In the paper of Kreyling et al. [28], after the intra-tracheal inhalation of NPs in rats, the TiO_2_ translocated across the air-blood barrier and were predominantly excreted in urine, whilst the translocated Au NP fraction and its excretion in urine was only negligible.

Regarding In_2_O_3_ NPs, a number of 14,020 particles/mL (1.00 ng/mL) was found in the blood of worker no. 2 while half of the particles in worker no. 3 (6722 particles/mL and 0.41 ng/mL) at diameters of ca. 38 nm (Table 4). No particles of In_2_O_3_ were observed in the worker no. 1 and in control samples. In line with this observation, Liu et al. [6] found that workers in the indium tin oxide (ITO) manufacturing department had serum levels of In significantly higher than the administrative staff. We cannot exclude that the obtained HBM results may be due to an inappropriate fitting of PPE during job procedures that could explain the differences in the levels of In_2_O_3_ NPs in blood of workers. However, for a correct interpretation of our findings, it seems important to consider that the cleaning activity of the CBE chamber for the growth of InAs nanostructures, as a possible condition of occupational exposure, is rarely performed (ca. twice a year).

In Company B, urinary and blood concentrations of Ir, Pd, and Pt were not detected in any subject (Table 3 and Table 4). These results showed that in the case of Company B, the collective and personal preventive measures adopted were effective in reducing the human exposure to these elements.

The effects of the ENPs analysed on human health are, to a large extent, unknown at present. Preliminary HBM information on occupationally exposed subjects reported possible changes in biomarkers of effect, e.g., oxidative stress, lipid peroxidation, and inflammatory, and also cardiovascular and respiratory biomarkers in workers exposed to ENPs including Ag, Au and TiO_2_, indium tin oxide (ITO) [29,30,31,32].

In addition, there no reference limits for ENPs in human biological samples derived by using the metric as described in this study, and thus the present data cannot be compared with any guideline. Notwithstanding this, some workplace guidelines have been developed by the World Health Organization (WHO) and the National Institute for Occupational Safety and Health (NIOSH) for the inhalation exposure to ENPs in workplaces [33,34]. These guidelines have been developed with the aim of protecting workers from the potential health risks to ENPs. In particular, the WHO reported the occupational exposure limit (OEL) values in air below which no adverse health effects will occur for specific ENPs including TiO_2_ and Ag, and NIOSH derived a recommended exposure limit (REL) for TiO_2_ in air.

Furthermore, the results suggested that different ENPs may have different toxico-kinetics. In fact, Ag NPs were measured in urine of enrolled workers whilst they were not found in their blood. Conversely, no particles of Au and TiO_2_ were found in urine, while they were detected in blood of both exposed and unexposed controls. This may be due to the diverse toxicokinetic behaviors among the various NPs, depending on the physicochemical properties (i.e., size), dissolution property in biological fluids, and NP–protein interaction, etc. [35]. Our survey showed that most of the particles (Au, In_2_O_3,_ and TiO_2_) were concentrated in the blood, whilst Ag was preferentially excreted via urine. Indeed, concentration of particles in the range of 0.41–4.9 ng/mL were found in blood while levels less than 0.01 ng/mL were found in urine (Figure 5).

Overall, the findings support the possibility to use the SP-ICP-MS methodology and the HBM approach to evaluate human exposure to ENPs in diverse occupational settings. However, the preliminary nature of these data and the limited number of subjects involved do not allow to extrapolate definitive conclusions on the interpretation of the results obtained.

## 5. Conclusions

Our study represents the first attempt to evaluate the occupational exposure to ENPs through a HBM survey and SP-ICP-MS analytical technique. Our results confirmed the exposure of workers to some nanosized-metals such as In_2_O_3_, although the limited number of subjects investigated. On the other hand, the comparable results between exposed workers and controls for Ag, Au, and TiO_2_ argued for a possible condition of extra-professional exposure. Even if these findings need to be confirmed in future studies, they preliminarily support the suitability of the SP-ICP-MS based methodology for the HBM of the ENP exposure in diverse occupational settings. Once confirmed on large workers populations, they may provide inputs to inform adequate strategies for ENP exposure assessment as a key issue of risk characterization. Overall, this may be also useful to define and update risk assessment and management procedures in nanomaterial companies to better protect the health of exposed workers. In addition, the proposed method may be applied also to assess the exposure to ENPs in the general population, to achieve knowledge on possible background levels of exposure, given the very low detection limits and the high accuracy and precision of the SP-ICP-MS technique.

## Figures and Tables

**Table 1:** Single Particle-Inductively Coupled Plasma-Mass Spectrometry (SP-ICP-MS) method parameters.

Instrument	ICAP-Q (Thermo Fisher)
Nebulizer	Quartz concentric
Spray chamber	Quartz cyclonic
RF power (W)	1400
Nebulizer gas flow (L/min)	1.02–1.06
Isotopes	^107^Ag, ^197^Au, ^115^In, ^192^Ir, ^106^Pd, ^195^Pt, ^47^Ti
Sample uptake rate (mL/min)	0.30
Acquisition mode, time (s)	Q-Cell in KED (4.8 mL/min He), 60
Transport efficiency (%)	4.5
Dwell time (msec)	5
Density (g/cm^3^)	Au, 19.3; Ag, 10.5; In_2_O_3_, 7.18; Ir, 22.4; Pd, 12.0; Pt, 21.4; TiO_2_, 4.23
Mass fraction (%)	Au, 100; Ag, 100; In, 83; Ir, 100; Pd, 100; Pt, 100; Ti, 60

**Table 2:** Single Particle-Inductively Coupled Plasma-Mass Spectrometry (SP-ICP-MS) validation study.

Metal NPs	Parameter (n = 5)	Urine	Blood
Ag 40 nm 0.05 ng/mL	Concentration LoD (ng/mL)	0.001	0.001
	Particle concentration spike recovery (%)	80.1	97.1
	Particle concentration spike repeatability (%)	0.4	1.1
	Size LoD (nm)	12.1	15.2
	Found diameter (nm) (RSD%)	32.9 (2.5)	33.2 (0.8)
Au 60 nm 19,000 particles/mL	Concentration LoD (particles/mL)	1063	1332
	Particle concentration spike recovery (%)	105.0	103.6
	Particle concentration spike repeatability (%)	4.5	5.1
	Size LoD (nm)	8.1	11.0
	Found diameter (nm) (RSD%)	66.1 (1.2)	72.0 (0.8)
In_2_O_3_ 20–70 nm 5.0 ng/mL	Concentration LoD (ng/mL)	0.36	0.40
	Particle concentration spike Recovery (%)	98.9	100.6
	Particle concentration spike repeatability (%)	3.9	3.3
	Size LoD (nm)	12.3	17.1
	Found diameter (nm) (RSD%)	42.8 (8.7)	45.4 (4.6)
Ir 15 nm 5.0 ng/mL	Concentration LoD (ng/mL)	0.76	1.02
	Particle concentration spike recovery (%)	98.7	96.5
	Particle concentration spike repeatability (%)	3.0	4.3
	Size LoD (nm)	6.6	7.8
	Found diameter (nm) (RSD%)	27.7 (8.2)	30.8 (8.4)
Pd 15 nm 5.0 ng/mL	Concentration LoD (ng/mL)	0.33	0.68
	Particle concentration spike recovery (%)	108.4	87.0
	Particle concentration spike repeatability (%)	3.6	4.2
	Size LoD (nm)	23.8	25.9
	Found diameter (nm) (RSD%)	32.9 (1.2)	35.4 (2.1)
Pt 15 nm 0.5 ng/mL	Concentration LoD (ng/mL)	0.07	0.13
	Particle concentration spike recovery (%)	83.6	105.8
	Particle concentration spike repeatability (%)	3.3	6.0
	Size LoD (nm)	16.1	17.3
	Found diameter (nm) (RSD%)	17.5 (7.0)	19.5 (5.7)
TiO_2_ < 100 nm 5.0 ng/mL	Concentration LoD (ng/mL)	0.28	0.81
	Particle concentration spike recovery (%)	99.3	99.1
	Particle concentration spike repeatability (%)	1.4	1.6
	Size LoD (nm)	45.2	50.2
	Found diameter (nm) (RSD%)	89.6 (2.8)	85.5 (4.6)

**Table 3:** Analysis of metal nanoparticles (NPs) in urine of workers from Company A (no. 1–3), Company B (no. 4–6) and controls (no. 7–8), at pre-shift (1st–4th day, mean) and at post-shift (1st–4th day, mean).

Metal NPs	Workers no. (Company)	Diameter ± SD (nm)		Number of particles ± SD (Particles/mL)		Concentration ± SD (ng/mL)	
		Pre-Shift	Post-Shift	Pre-Shift	Post-Shift	Pre-Shift	Post-Shift
Ag	1–2 (A)	nd	nd	nd	nd	Nd	nd
	3 (A)	29.2 ± 2.9	29.4 ± 2.5	19,180 ± 9921	13,955 ± 4608	0.012 ± 0.005	0.009 ± 0.002
Au, In, Pt, Ti	1–3 (A)	nd	nd	nd	nd	nd	nd
Ir, Pd, Pt	4–6 (B)	nd	nd	nd	nd	nd	nd
**Metal NPs**	**Controls no.**	**Diameter ± SD** **(nm)**		**Number of Particles ± SD** **(Particles/mL)**		**Concentration ± SD** **(ng/mL)**	
		**Pre-Shift**	**Post-Shift**	**Pre-Shift**	**Post-Shift**	**Pre-Shift**	**Post-Shift**
Ag	7	28.9 ± 1.9	28.4 ± 2.5	7680 ± 12,327	6630 ± 4087	0.006 ± 0.006	0.006 ± 0.002
	8	28.6 ± 2.1	28.2 ± 2.6	12,215 ± 4916	10,470 ± 396	0.008 ± 0.002	0.007 ± 0.002
Au, In, Ir, Pd, Pt, Ti	7–8	nd	nd	nd	nd	nd	nd

nd, not detected; SD, standard deviation.

**Table 4:** Analysis of metal nanoparticles (NPs) in blood of workers from Company A (no. 1–3), Company B (no. 4–6) and controls (No. 7–8), at the end of working week (4th day).

Metals NPs	Workers no. (Company)	Diameter ± SD (nm)	Number of Particles (Particles/mL)	Concentration (ng/mL)
Ag	1–3 (A)	nd	nd	nd
Au	1 (A)	15.3 ± 2.3	251,177	nd
	2 (A)	15.5 ± 2.5	62,794	nd
	3 (A)	15.1 ± 2.2	65,934	nd
In	1 (A)	nd	nd	nd
	2 (A)	37.9 ± 1.6	14,020	1.00
	3 (A)	37.6 ± 1.3	6722	0.41
Pt	1–3 (A)	nd	nd	nd
Ti	1 (A)	82.4 ± 12.5	49,092	4.90
	2 (A)	83.4 ± 13.3	16,709	1.87
	3 (A)	86.8 ± 14.8	17,315	1.93
Ir, Pd, Pt	4–6 (B)	nd	nd	nd
**Metals NPs**	**Workers no.** **(Company)**	**Diameter ± SD** **(nm)**	**Number of Particles** **(Particles/mL)**	**Concentration** **(ng/mL)**
Ag	7–8	nd	nd	nd
Au	7	15.1 ± 1.5	10,989	nd
	8	15.3 ± 2.2	95,838	nd
In, Ir, Pd, Pt	7–8	nd	nd	nd
Ti	7	84.5 ± 12.0	16,245	1.83
	8	90.4 ± 16.4	14,334	1.65

nd, not detected. SD, standard deviation.

## Data Availability

Not applicable.
